# Supplementary figures and images for: Systematic identification of functional SNPs interrupting 3’UTR polyadenylation signals
Source: PLoS Genet. 2020 Aug 17;16(8):e1008977. doi: 10.1371/journal.pgen.1008977 (PMC7451987; doi:10.1371/journal.pgen.1008977)

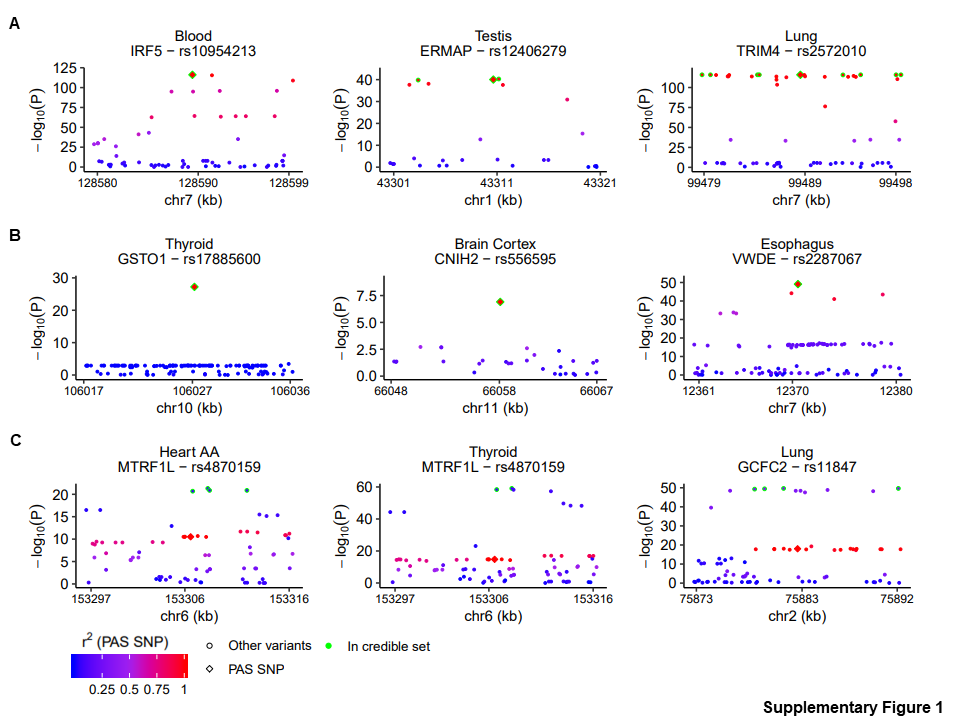

Supplement: S1 Fig — A. The credible sets that were defined by CAVIAR for the pA loci presented in Fig 2B–2D. B. Examples for PAS SNPs with very high posterior probability pointing them as the causal pA-QTL variants with very high confidence. C. Examples of PAS SNPs that obtained a significant p-value in the pA-QTL tests, but were not included in the credible sets of their pA loci, indicating that they are not the causal ones. Varaints included in the credible set are colored in green. Otherwise, the legend is as in Fig 6. (TIF) [file pgen.1008977.s001.tif]

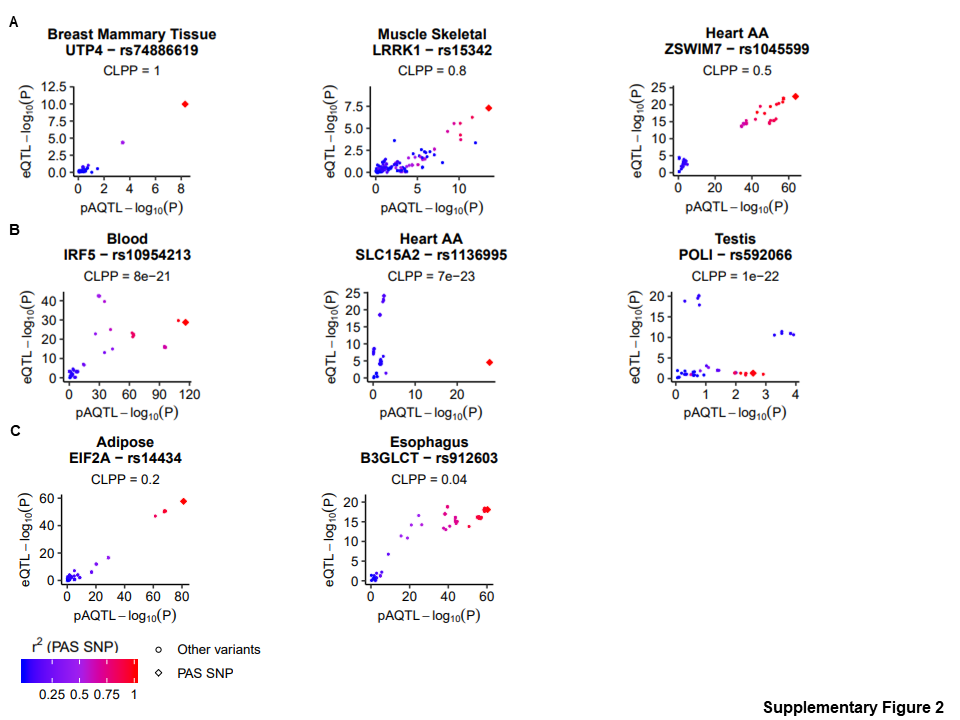

Supplement: S2 Fig — A. Examples of loci showing strong colocalization of the pA-QTL and eQTL signals (very high colocalization posterior probability (CLPP)), indicating the PAS SNP as the causal variant for both effects. B. Examples of weak colocalization of the pA-QTL and eQTL signals (low CLPP), suggesting that the eQTL tags a causal variant that is distinct from the PAS SNP. C. Strong colocalization of the pA-QTL and eQTL signals in the loci shown in Fig 5A and 5B. Legend is as in Fig 6. (TIF) [file pgen.1008977.s002.tif]

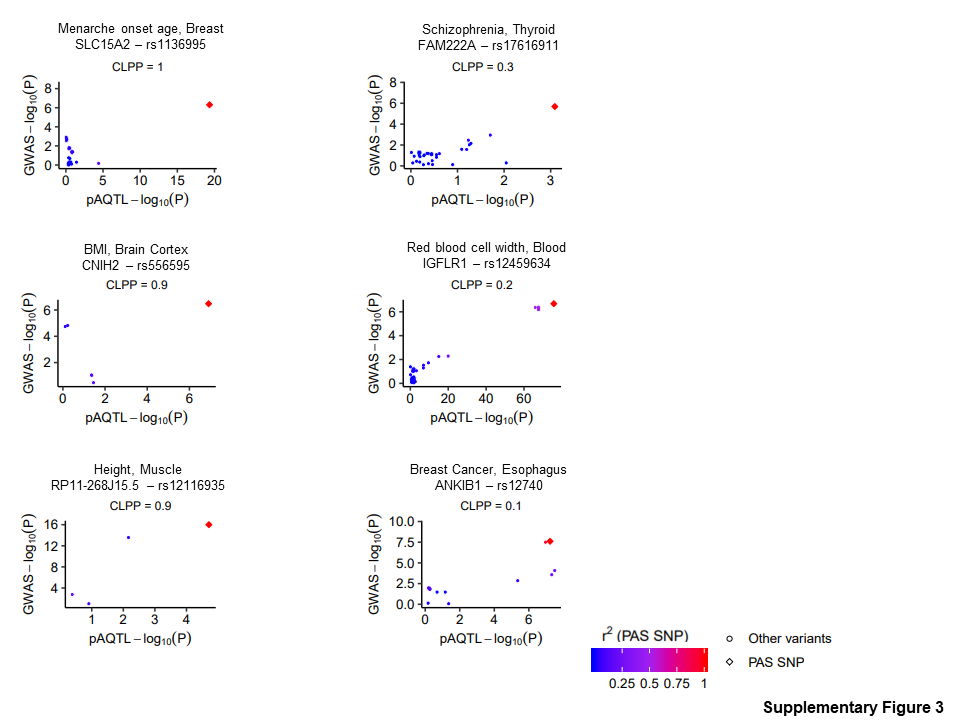

Supplement: S3 Fig — Examples of PAS pA-QTLs and GWAS SNPs showing high colocalization (CLPP>0.01), indicating a common underlying causal mechanism. Legend is as in Fig 6. (TIF) [file pgen.1008977.s003.tif]

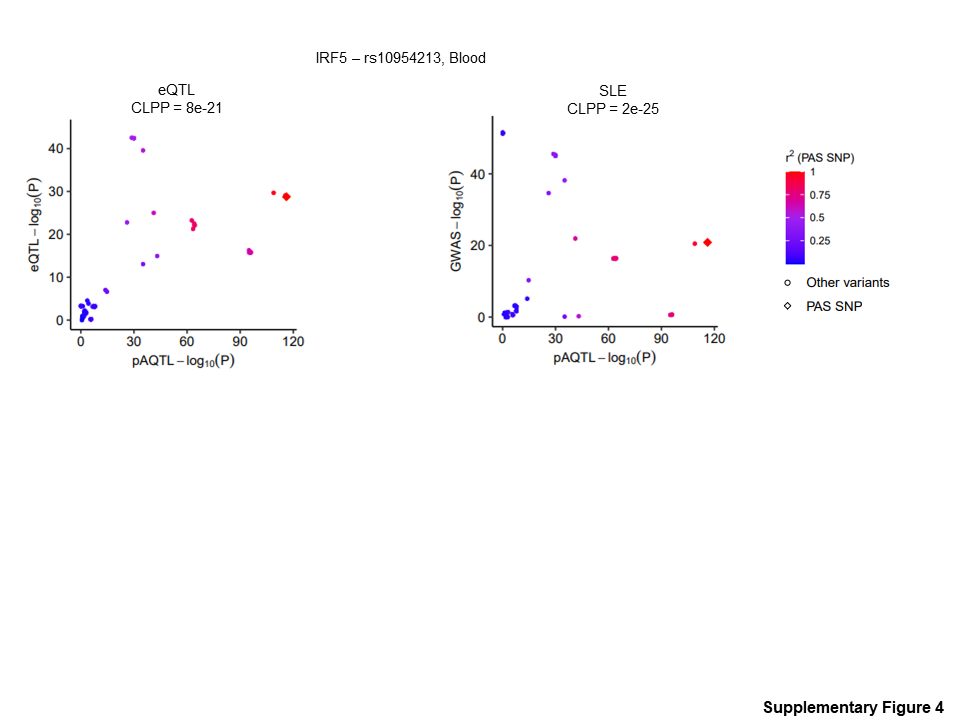

Supplement: S4 Fig — Legend is as in Fig 6, See S6 Table. (TIF) [file pgen.1008977.s004.tif]

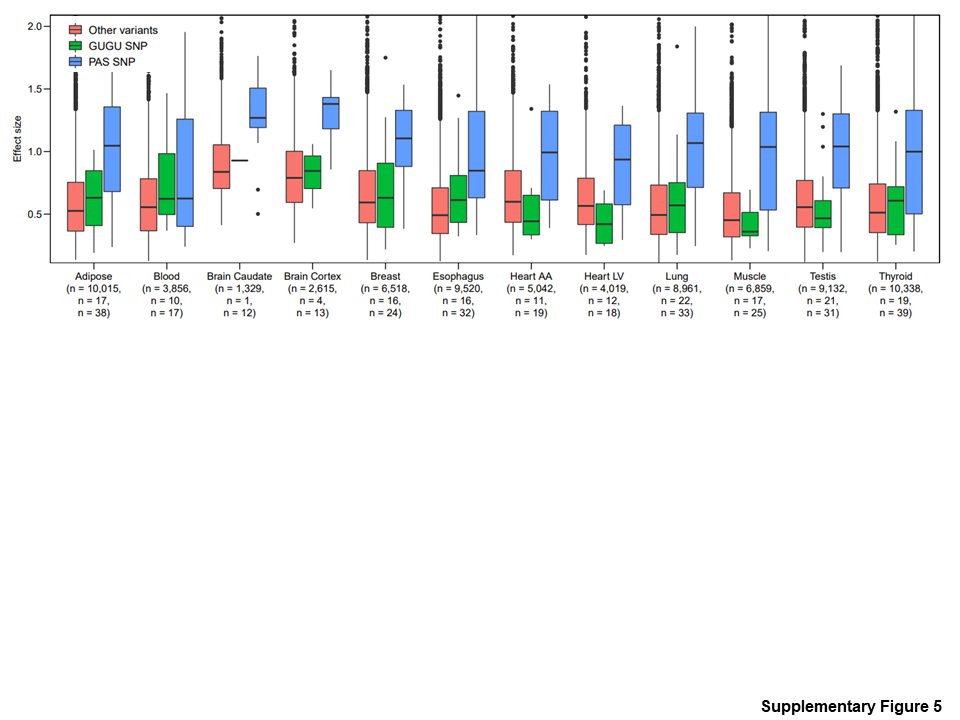

Supplement: S5 Fig — Axis and legend as Fig 7A. (TIF) [file pgen.1008977.s005.tif]

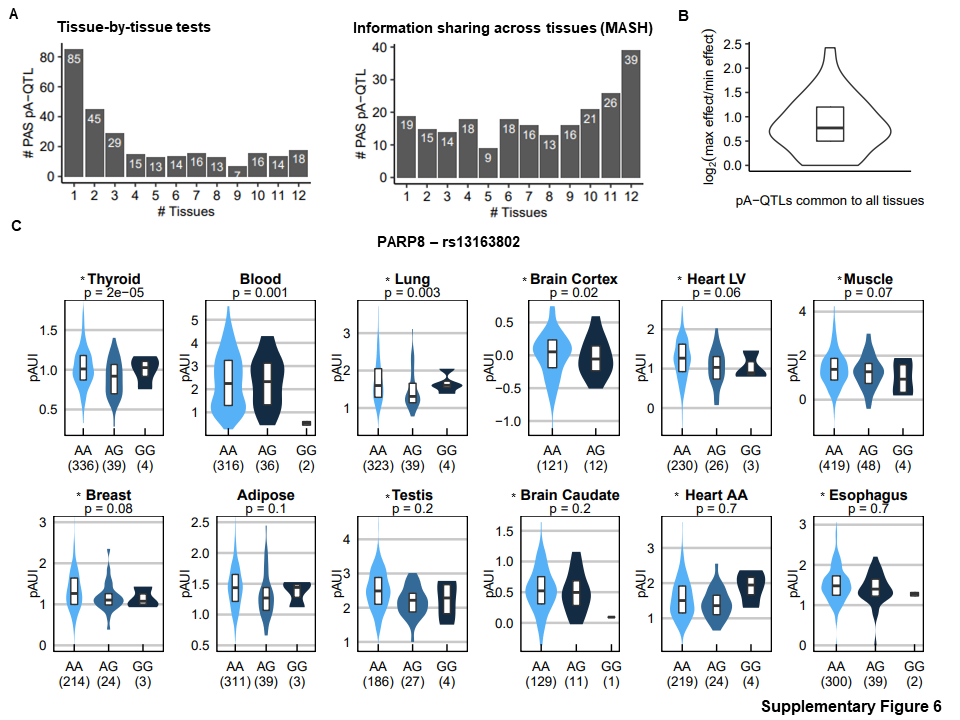

Supplement: S6 Fig — A. Distribution of the number of different tissues in which each PAS SNP pA-QTL was detected by the tissue-by-tissue tests (left) and by MASH analysis utilizing information sharing across tissues (the analysis shown here includes variants that were tested (that is, passed the filtering criteria) in all tissues). The proportion of PAS SNPs that were detected as pA-QTLs in at least nine tissues was up from 20% in the tissue-by-tissue tests to 45% in MASH analysis. B. PAS SNPs with a significant effect in all twelve tissues (39 SNPs) show high variation in their effect sizes over the tissues. For each PAS SNP detected by MASH as a pA-QTL in all tissues, we calculate the ratio between its maximal and minimal effect size (MASH posterior mean). The violin plot shows the distribution of these ratios. A third of these pA QTLs show more than a 2-fold difference in their effect size. C. An example of a PAS pA-QTL that reached statistical significance in only two tissues (Thyroid and Blood) by the tissue-by-tissue FastQTL tests, and turned significant in ten tissues by MASH analysis based on information sharing across tissues. (The indicated p-values are those obtained by the tissue-by-tissue FastQTL tests, asterisks next to the tissue name indicate pA-QTL according to MASH analysis). (TIF) [file pgen.1008977.s006.tif]
