# Supplementary material for: Systematic identification of functional SNPs interrupting 3’UTR polyadenylation signals
Source: PLoS Genet. 2020 Aug 17;16(8):e1008977. doi: 10.1371/journal.pgen.1008977 (PMC7451987; doi:10.1371/journal.pgen.1008977)
Supplement: Attachment — Submitted filename: pAQTL_response_letter.pdf [file pgen.1008977.s013.pdf]

March 15, 2020

Dear Editor,

First, we would like to thank the referees for their time and effort in reviewing our manuscript. The referees' comments were very constructive and our study benefited greatly from them. We submit a revised version of the manuscript which addresses all the points raised by the referees as detailed below, point-by-point.

Sincerely,

Ran Elkon, Ph.D.

Department of Human Molecular Genetics & Biochemistry
Sackler School of Medicine
Tel Aviv University

Reviewer #1: *The manuscript "Systematic identification of functional SNPs interrupting 3' UTR polyadenylation signals" by Shulman and Elkon examines genome-wide, the effect of SNPs that interrupt the polyadenylation signal (PAS) in the 3' UTR on differential poly(A) site usage using RNA-seq data from 12 tissues in GTEx through QTL analysis (pA-QTL) and their relationship with complex disease or trait associations. The authors focus on variants that fall in canonical PAS within 40 nucleotides of the poly(A) tail, which are expected to have the strongest effects on polyA. They find 139 significant pA-QTLs, half of which overlap with expression QTLs (eQTLs), and 53 of which are in linkage disequilibrium (LD) with complex disease or trait associations, proposing a new (under investigated) molecular mechanism that may underlie genetic associations with complex diseases/traits. One finding the authors find unexpected is that 12% of variants in the polyA motif that lead to 3' UTR lengthening are associated with increased expression of the gene, while 88% of cases are associated with decrease in expression. Transcript lengthening has primarily been thought to have a destabilizing effect.*

This work presents a first systematic, genome-wide examination of variants in PAS association with differential polyadenylation levels in various tissues, providing new insights on the effect of genetic variation on transcript expression and human complex traits. While I think such a resource would be very valuable to the genetic community, I have several technical comments/concerns and suggestions on the analyses which I think need to be addressed for the results to be sound and more broadly applicable.

<u>Major comments</u>:

1. The authors performed QTL (association) analysis between SNPs found in polyadenylation signals (PAS) within 40 nucleotides of annotated 3' UTR polyadenylation (p(A)) sites and p(A) site usage based on reads that map to the

common 3' UTR segment (upstream of the pA site) relative to the reads that map to the alternative 3' UTR segment (downstream of the pA site). While this is a first obvious region to inspect for genetic effects on polyadenylation, as disruption of the canonical PAS has been shown to have strong effect on pA usage, there may be other cis effects on polyadenylation (e.g., binding of RNA-binding protein to the DNA). I think it would be informative to test for genetic associations with pA usage considering also variants further away from the 3' UTR, e.g., within +/-1Mb of the pA site (this would identify other types of mechanisms that may affect pA usage).

Our original analysis was confined to variants disrupting the canonical PAS as these are expected to have the strongest effect on APA. Indeed, there are many more variants likely to affect APA through both direct and indirect mechanisms. Direct effects are mainly mediated by cis-regulatory elements in 3'UTRs (auxiliary APA motifs, RBP target sites) and indirect effects are probably mediated by variants affecting transcription and splicing (as both processes have been shown to be coupled with APA). In the revised version, we substantially extended our pA-QTL analysis but still opt to focus the current study on putative direct effects on APA. Therefore, in the revised ms, after the sections focused on PAS SNPs, we include an extended pA-QTL analysis that was applied to all 3'UTR SNPs (page 8, Figure 7A). As expected, this analysis discovered thousands of additional pA-QTLs, having weaker effects than the PAS SNPs.

2. Also, in computing pA site usage, did the authors apply a lower bound cutoff of number of reads that map to the 3' UTR or lower bound density of reads per Kb?

Our revised analysis first followed GTEx v7 analysis and included only genes with > 0.1 TPM and ≥ 6 reads in 20% of the samples. In addition, we required that 3'UTRs have a median RPKM ≥ 1 across the tissue's samples.

3. If a PAS SNP had more than one annotated p(A) site within downstream 40 nucleotides, the nearest pA site was taken. Why not test all adjacent pA sites and correct for this in the permutation analysis when assessing significance of associations per gene?

PAS elements are typically located ~20-25 nt upstream their target p(A) sites. To increase coverage we broadened the window to 40 nt. It is indeed quite common that cleavage loci show clustering of very close p(A) sites (typically with distance below 10 nt). Our pA-QTL analysis is based on the pAUI index (calculated as the ratio between reads mapped to the cUTR and aUTR intervals). We did not test the effect of a PAS SNP on all its adjacent p(A) sites separately, as their pAUI is essentially the same.

4. There is no mention of adding hidden expression covariates (e.g., PEER factors, Stegle et al., 2012) to the linear regression model of the pA-QTL analysis. It is very important to add expression covariates, as for example the first expression PEER factors in GTEx are highly correlated with ischemic time that affect RNA degradation, which is a factor that can affect the pA usage, or cellular composition heterogeneity. The PEER factors are provided on the GTEx portal (https://www.gtexportal.org/home/) for all tissues.

Thank you for this comment. As described in the Methods section, our revised pA-QTL analysis now includes also PEER factors (obtained from GTEx) as covariates.

5. For the pA-QTL analysis the authors use a minor allele frequency cutoff of >0.1. At the sample size of over 130 samples there is power to detect associations at a minor allele frequency of 0.05.

Thanks. This was a typo. We followed GTEx v7 criteria and used MAF>0.01 in addition to requiring that the minor allele is observed in at least 10 samples.

6. Can the authors expand on how they computed q-values from the QTL association p-values? Is this using permutation analysis or fitting a beta distribution? Was the FDR applied at the gene level, e.g. taking the best signal per gene? A description of how to properly account for differences in number of variants tested per gene and local LD can be found in GTEx v6 paper (PMID: 29022597) or on the GTEx portal.

Our original analysis included a single SNP per 3'UTR (so multiple testing considered the number of distinct 3'UTRs tested). Our revised analysis is significantly extended and considers numerous variants per 3'UTR (those within +/-10kbp with respect to p(A) sites for the PAS pA-QTL analysis; and those within 3'UTRs +/- 2.5 kbp flanks for the general 3'UTR pA-QTL analysis). This revised analysis is done using FastQTL and GTEx v7 methodology for multiple testing correction. Details are provided in Methods. Briefly, empirical p-values per 3'UTR were modeled by a Beta distribution which reports a p-value of the most significant variant. These p-values were further corrected for multiple testing (due to testing numerous 3'UTRs) using Storey's q-value method. Additionally, we implemented the methodology of GETx for calculating a nominal p-value threshold per pA locus.

7. To inspect the connection between the effect of the pA-QTLs and genetic associations with complex human diseases and traits, the authors test for overlap/co-occurrence of signals. However, co-occurrence of QTL and GWAS signals may just be by chance, given the clustering of GWAS signals around genes and their high abundance (PMID: 31043754). Bayesian-based colocalization methods have been developed to test whether two co-occurring association signals are tagging the same causal variant or haplotype, such as eCAVIAR (PMID: 27866706) and ENLOC (PMID: 28278150) which consider local allelic heterogeneity. I would recommend applying such methods to gain higher confidence in the potential causal effect of pA-QTLs on complex human traits as for example done in GTEx release v8 preprint (https://www.biorxiv.org/content/10.1101/787903v1).

Thank you so much for raising this critical issue. In the revised manuscript we now use eCAVIAR to test for colocalization between pA-QTL and GWAS signals (and eQTL signals, as recommended below).

* Also, it would be informative to report the direction of effect of pA-QTLs on disease risk or trait variation for those pA-QTLs that colocalize with GWAS associations? Does lengthening or shortening of the transcript tend to be associated with increased risk of disease?

We added an indication for the direction of the effect of the PAS-interrupting allele on the disease/trait (Supplementary Table 3).

8. To test whether the 139 pA-QTLs were also significant eQTLs, the authors tested whether the pA-QTL variants were also significant eQTLs. However, similarly to GWAS associations, pA-QTL and eQTL signals may be overlapping but tagging different causal variants. To further support that the pA-QTL and are also eQTLs, I would also recommend applying colocalization analysis between co-occurring pA-QTLs and eQTLs,

As indicated above, in the revised manuscript, we used eCAVIAR also to test for colocalization of pA-QTL and (GTEx) eQTL signals.

9. To better estimate sharing of pA-QTLs across tissues, there are multi-tissue methods that account for effect size and standard error across tissues, to increase power in each tissue. The multivariate adaptive shrinkage method, MASH (PMID: 30478440) is one such method that estimates tissue sharing and specificity of genetic associations.

Thank you for this suggestion. In the revised version, we applied MASH and show the benefit of information sharing across tissues (results shown in Supplementary Figure 6).

<u>Minor comments</u>:

1. 139 PAS SNPs were found to be significant pA-QTLs at FDR<5%. For how many genes? Was only one variant association found to be significant per gene?

Our revised analysis detected, overall, 330 distinct PAS pA-QTLs (FDR<5% and included in CAVIAR credible set). These PAS SNPs are located in 324 distinct genes (3'UTRs).

Reviewer #2: *This is a nicely carried out work on SNP impacts on APA. This is also a timely piece of work, because several studies are reporting similar findings. One key finding of this work is the relationship between 3'UTR size control and gene expression. I have only a few minor comments*:

1. Have the authors tried a different window for SNP analysis than 40 nt? It would be helpful to readers if they can show SNPs in more upstream or downstream regions.

In the revised manuscript we significantly expanded our pA-QTL analysis, and we include a section that discusses the results we obtained when testing variants (which passed our filtering) located anywhere in the 3'UTR (+/- 2.5 kbp flanks) of >22k genes.

2. A sizable fraction of APA-affecting SNPs do not involve canonical signals. The authors need to show some of the samples and discuss them.

We thank there referee for this suggestion. We now discuss a subset of pA-QTLs that seem to affect APA by disruption of GUGU motifs, located downstream of the respective p(A) site (Figure 7).
