# Supplementary material for: Systematic identification of functional SNPs interrupting 3’UTR polyadenylation signals
Source: PLoS Genet. 2020 Aug 17;16(8):e1008977. doi: 10.1371/journal.pgen.1008977 (PMC7451987; doi:10.1371/journal.pgen.1008977)
Supplement: Attachment — Submitted filename: Response_letter_rev2.pdf [file pgen.1008977.s014.pdf]

May 7, 2020

Dear Editors,

First, we would like to express our sincere gratitude to you and the referees for your time and effort in reviewing our revised manuscript during the difficult period of the COVID19 outbreak. We were heartened to see that both the editors and referees appreciated the analyses we added to the revised version and the overall significance of our findings. We hereby submit an updated version of the manuscript which addresses the last remaining minor points, as detailed below, point-by-point.

Sincerely,

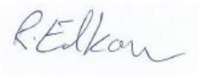

`

Ran Elkon, Ph.D.

Department of Human Molecular Genetics & Biochemistry
Sackler School of Medicine
Tel Aviv University

Reviewer #1: The authors of the manuscript “Systematic identification of functional SNPs interrupting 3’ UTR polyadenylation signals” have done a lot of work to the revised version and have carefully addressed all of my comments/concerns. I only have a few remaining minor comments:

1. To test whether the significant pA-QTL colocalize with GWAS signals, the authors tested 124 complex diseases and traits with available summary statistics. They tested for significant colocalization between PAS pA-QTL with GWAS p-values below 1E-05 (Methods, page 13). Given the multiple hypothesis burden of testing millions of common variants in GWAS, the cutoff P<5E-08 has been typically used as the genome-wide significant cutoff. I would recommend to distinguish between GWAS loci whose lead variant passes genome-wide significance and those that are subthreshold associations (5E-08).

Thank you for this suggestion. We now distinguish between these two cases in TabS3 and in the relevant text. We also found a mistake in the number we reported in the previous version for the overlap between PAS pA-QTLs and GWAS SNPs. The correct number is now reported (104 SNPs (instead of 153), 70 of which have GWAS p-value <5E-08) (page 7 – ‘Colocalization of PAS pA-QTL and GWAS signals’ section).

2. On the top of page 6, the authors point out that they did not capture the known association between the PAS SNP in the 3'UTR of IRF5 and risk for SLE when setting the eCAVIAR parameter for number of putative causal variants to 1, “because this locus likely contains several causal variants that act through different mechanisms, some of which have a stronger effect on IRF5 expression and SLE risk than the PAS pA-QTL (S4 Fig).” This indeed seems to be the case. The authors go on to test this by setting the parameter to multiple potential causal variants in the SLE GWAS and eQTL loci, and report that then they do find significant colocalization with 3-5 putative causal variants. Can the authors add these results to a supplementary table? What are the posterior probabilities of the lead GWAS and pA-QTL variants?

These results are now provided (TabS5).

3. On the bottom of page 5, in the sentence: “Overall, 512 pA sites were significantly associated (FDR=5%) with a pA-QTL in at least one tissue (see examples in Fig 2B-D; S3 Table).” it should be written FDR<=5%, not (FDR=5%).

Thanks. Corrected.

4. On the bottom of page 6, the authors report the results of testing for colocalization of co-occurring PAS pA-QTLs and eQTLs, also in Table S3: “Of the PAS pA-QTLs that overlapped an eQTL, 51 showed a high colocalization in at least one tissue (S3 Table).” I think it would be helpful to generate a table that summarizes just these 51 significantly colocalizing results, to be able to easily view these results over the thousands of tests performed across all tissues.

We now provide the PAS pA-QTLs in ranked order in TabS3, such that those that showed significant colocalization with eQTLs are at the top of the list (sorted by the number of tissues colocalization was found).

5. On the top of page 7 and in Figure 5C, the authors note that 3'UTR lengthening in all tissues is significantly associated with decreased expression of the target gene. Is this for all overlapping pA-QTL and eQTL or just for the 51 significantly colocalizing loci? If the latter, it would be clearer to write ‘Colocalizing pA-QTL / eQTL loci’ in the x-axis of Fig. 5C.

This analysis (Fig 5C) includes all overlapping pA-QTL and eQTL PAS SNPs. It shows the consistency of the effect of 3’UTR shortening on gene expression even in cases where significant colocalization was not detected by eCAVIAR (and supporting an effect for these SNPs on expression also in these cases).

6. In figures 5D and 7D, arrows that indicate the direction of the link between the PIA and gene expression were added only to a subset of the genes by tissue squares. Were they added only to significantly colocalizing pA-QTL/eQTL loci? If so, I would recommend clarifying this in the figure legends.

In these figures, arrows were added all overlapping pA-QTLs and eQTLs. Significant colocalization is indicated by the darker color of these cases. This is clarified in the legend in

both the text and the figure itself. To further clarify this point, we added to the legend that squares without an arrow signify no overlapping eQTL.

7. On the top of page 6, I would add the words “per tissue” at the end of the following sentence: “Defining per pA site with a significant association a 95% credible set of variants, this analysis implicated the PAS SNP as a causal variant for 55%-70% of the observed pA-QTLs (Fig3A).”

Indeed. Thanks. We added “per tissue” to this sentence.

8. In the Methods section on the bottom of page 12 and on pages 13 and 14, the authors use the term ‘t-score’ to represent the regression slope divided by its standard error. I think it is more correct to call this value ‘t-statistic’.

Thanks. Corrected.

9. There is a typo on the bottom of page 13: ‘GETx’ should be ‘GTEx’.
There is a typo on the top of page 14: -keep alle-order should be ‘-keep allele-order’.
There is a typo in the word ‘slop’ (should be ‘slope’) in the MASH paragraph in the Methods section on the bottom of page 14.

We apologize for these typos and thank the referee for spotting them. They are now corrected.

Reviewer #2: The authors have addressed all my concerns.
